# Dazomet changes microbial communities and improves morel mushroom yield under continuous cropping

**DOI:** 10.3389/fmicb.2023.1200226

**Published:** 2023-08-08

**Authors:** Bo Chen, Gaige Shao, Tao Zhou, Qinghao Fan, Nuolin Yang, Man Cui, Jinwei Zhang, Xiangli Wu, Bangxi Zhang, Ruiying Zhang

**Affiliations:** ^1^Institute of Soil and Fertilizer of Guizhou Province, Guiyang, China; ^2^Xi'an Agricultural Technology Extension Center, Xi'an, China; ^3^Fruit and Vegetable Workstation of Guizhou Province, Guiyang, China; ^4^State Key Laboratory of Efficient Utilization of Arid and Semi-arid Arable Land in Northern China, Institute of Agricultural Resources and Regional Planning, Chinese Academy of Agricultural Sciences, Beijing, China

**Keywords:** morel mushroom, *Morchella sextelata*, dazomet, microbial community, continuous cropping obstacle

## Abstract

Morels (*Morchella* spp.) are highly prized and popular edible mushrooms. The outdoor cultivation of morels in China first developed at the beginning of the 21st century. Several species, such as *Morchella sextelata*, *M. eximia*, and *M. importuna*, have been commercially cultivated in greenhouses. However, the detriments and obstacles associated with continuous cropping have become increasingly serious, reducing yields and even leading to a complete lack of fructification. It has been reported that the obstacles encountered with continuous morel cropping may be related to changes in the soil microbial community. To study the effect of dazomet treatment on the cultivation of morel under continuous cropping, soil was fumigated with dazomet before morel sowing. Alpha diversity and beta diversity analysis results showed that dazomet treatment altered the microbial communities in continuous cropping soil, which decreased the relative abundance of soil-borne fungal pathogens, including *Paecilomyces*, *Trichoderma*, *Fusarium*, *Penicillium*, and *Acremonium*, increased the relative abundance of beneficial soil bacteria, including *Bacillius* and *Pseudomonas*. In addition, the dazomet treatment significantly increased the relative abundance of morel mycelia in the soil and significantly improved morel yield under continuous cropping. These results verified the relationship between the obstacles associated with continuous cropping in morels and the soil microbial community and elucidated the mechanism by which the obstacle is alleviated when using dazomet treatment.

## 1. Introduction

True morels (*Morchella* spp., Morchellaceae, Pezizales) are commercially important edible mushrooms with economic and scientific value. They are very popular in most of Asia, Europe and North America due to their delicious taste, special flavor, high nutritional value, low calorific value, and potent health-promoting abilities (Wu et al., 2021). The morels are characterized by the unique honeycomb appearance of the hollow fruiting body. In nature, wild morels are widely distributed in temperate regions of the Northern Hemisphere, and form fruiting bodies in the spring (Liu et al., 2018).

Over the centuries, various methods of morel cultivation have been attempted. Indoor cultivation of morel first developed in the early 1980s in the United States. In brief, sclerotia are first formed in spawn bags; then, sclerotia are used as spawn to inoculate composted substrates; and finally, fruiting bodies are induced by irrigation (Ower, 1982; Ower et al., 1986, 1988, 1989; Masaphy, 2010; Longley et al., 2019). Because of poor stability and repeatability, this technology has not been widely promoted and commercialized. Even so, this work was still a tremendous breakthrough in the history of morel cultivation.

The outdoor cultivation of morel first developed in China at the beginning of the 21st century. In short, the spawn is composed of vegetative mycelia rather than sclerotia and is inoculated in soil in the greenhouse environment in autumn. When conidia are formed on the soil surface, exogenous nutrient bags are placed in the field to promote the growth of the mycelia. In the early spring of the next year, the vegetative mycelia are stimulated by irrigation to promote differentiation and the formation of primordia (Tan, 2016; Liu et al., 2018). The most important breakthrough in outdoor cultivation has been the invention and application of exogenous nutrient bags. At present, a few morel species, including *Morchella sextelata*, *M. eximia*, and *M. importuna*, etc., have been commercially cultivated using outdoor cultivation techniques in China (Masaphy, 2010; Kuo et al., 2012; Loizides, 2017; Liu et al., 2018). This outdoor cultivation technology has been rapidly and commercially applied on a large scale. In 2020, the annual production of morels in China reached 15,000 tons (fresh weight) and continues to maintain a rapid growth trend (Yu et al., 2022).

In recent years, with the continuous expansion of morel cultivation in China and around the world, outdoor cultivation has often suffered from severe yield reduction and even a failure of fruiting bodies to be produced for unknown reasons (Sambyal and Singh, 2021; Tan et al., 2021; Yu et al., 2022). Some studies have shown that possible reasons leading to the failure of morel fructification include unstable quality of spawn, improper field management practices, unfavorable environmental factors, pathogens, soil characteristics, soil microbial community dynamics, etc. (Zhao et al., 2016; He et al., 2017, 2018; Lan et al., 2020; Wang et al., 2020; Tan et al., 2021; Yu et al., 2022). In fact, the obstacles associated with continuous cropping is also an important reason for the decline in morel yield. The continuous cropping obstacle refers to the phenomenon in which the same crop or a related species is continuously planted in the same plot and the yield and quality are reduced over time, even under normal management conditions (Chen Y. et al., 2022). Many crops, whether perennial or annual, suffer from continuous cropping obstacles (Li et al., 2016). Most edible mushrooms cultivated in farmland soil, such as *Ganoderma lucidum* and *Dictyophora* spp., have also been severely influenced by continuous cropping obstacles (Zhang et al., 2018; Jiang et al., 2021; Lu et al., 2022). The rotation of morel mushrooms and paddy rice is an effective measure to reduce or eliminate this obstacle. However, the application of this crop rotation is limited because most morel is cultivated in dryland greenhouses. To circumvent continuous cropping obstacles, the farmland used for morel cultivation must be changed every year, which greatly increases the cost of morel cultivation. The mechanisms of most continuous cropping obstacles are very complex and are associated with autotoxicity, deterioration of the physicochemical properties of soil, accumulation of soil-borne pathogens, and disruption of the soil microbial community (Xi et al., 2019). To date, the mechanism of continuous cropping obstacles in morel cultivation is not clear.

In recent years, disinfection of soil with fumigants before planting has been an effective and reliable method to prevent and control continuous cropping obstacles related to the soil microbial community (Li Q. et al., 2021). Methyl bromide was once the most commonly used fumigant. However, it has been banned because it destroys the ozone layer. Dazomet is a recently developed broad-spectrum soil fumigant that is usually used to control soil pests, weeds and pathogens in greenhouses, seedling farms and orchards. When dazomet is applied to wet soil, it degrades rapidly and releases methyl isothiocyanate (MITC), formaldehyde, monomethylamine and hydrogen sulfide. MITC is the main active substance. The nonspecific toxicity of MITC is due to its ability to cause perturbation of metal enzymes or thiol-containing proteins, its formation of reactive oxygen species or the metal toxicity it causes due to the chelation of heavy metals (Tilton et al., 2006; Consolazio et al., 2019). The soil retention time of dazomet is short, making it more environmentally benign. With the elimination of methyl bromide, dazomet has been registered in many countries. It has been reported that dazomet has been used in strawberry, tomato, flowers, ginger, cucumber, and other high-value crops (Chen R. et al., 2022). Dazomet impacts both beneficial and harmful soil organisms because it is broad-spectrum, but the severity of the impact varies according to the soil conditions and the species (Wang et al., 2011).

It has been reported that the obstacle associated continuous cropping in morels may be related to changes in the soil microbial community (Liu et al., 2022). To the best of our knowledge, the application of dazomet in the cultivation of edible mushrooms has not been reported. To study the effect of dazomet treatment on the cultivation of morel under continuous cropping, the soil was planted continuously for 1 year and 2 years was fumigated with dazomet before sowing. The growth, fructification, and yield of morel were investigated, and dynamic changes in bacterial and fungal communities were monitored using high-throughput gene sequencing. The results suggested that the dazomet treatments were effective in increasing the yield of morel by changing the microbial community under continuous cropping.

## 2. Materials and methods

### 2.1. The morel strain and medium

The experiments were carried out with the *M. sextelata* Qian-Morel 1 cultivar. The spawn and exogenous nutrient bags were provided by Guizhou Lefeng Biotechnology Co., Ltd. The spawn medium consisted of 85% wheat, 5% rice husk, 8% humus, 1% lime and 1% gypsum, with a moisture content of 60%. The medium in exogenous nutrient bags consisted of 89% wheat, 10% rice husk, and 1% lime, and the water content was 60%.

### 2.2. Experimental design

To test the effect of dazomet fumigation on the continuous cropping obstacle of morel, two experimental sites were selected in Guizhou Province, one of which was located in Tianba Town, Qixingguan District, and had been planted for 1 years. The other was in Tailai Township, Qianxi City, which had been planted for 2 years. The controls without fumigation and dazomet treatment were established on randomly designed plots, with three replicates for each treatment at each experimental site. The field was plowed with a rotary tiller to a depth of 25 cm. The relative humidity of the soil was adjusted to 65%. The dosage of dazomet (Synwill Co., Ltd) was 60 g/m^2^. The dazomet was sprinkled onto the surface of the soil and then evenly mixed with the soil using a small rotary tiller. The field was immediately covered with PE film to maintain the fumes of dazomet in the soil. After 20 days, the film was removed, and the greenhouse was kept ventilated for 15 days. Three independent biological replicates with 200 m^2^ each were conducted.

### 2.3. The cultivation of the morel

The morel was sown on the 35th day after fumigation, and the dosage of spawn was 300 g/m^2^. Seven days after sowing, morel mitospores, which was previously considered as asexual conidia (Liu et al., 2023), appeared on the soil surface. On the 10th day, exogenous nutrient bags punched on the side that would be in contact with the soil were placed on the soil surface. Five exogenous nutrient bags were placed per square meter, each containing 400 g of medium. The relative humidity of the soil was maintained at approximately 60%. The exogenous nutrient bags were removed on the 85th day after sowing. The field was irrigated to induce the formation of the primordia. The fruiting bodies were harvested on the 110th day after sowing.

The weight of morels harvested was converted to kg/m^2^ according to the area of each replicate. The yield was analyzed using SPSS (version 26.0) through ANOVA. *P* < 0.05 was used as the threshold.

### 2.4. Soil sampling

To investigate the changes in the soil microbial community, soil samples were collected from the experimental site in Tailai Township, Qianxi City. The soil samples were taken from a depth of 3–5 cm before sowing, at the primordial stage, and before harvest of the fruiting body (Table 1).

**Table 1:** Soil samples used in this study.

Soil sample	Stage of morel cultivation
SCK	Before sowing of the control without fumigation with dazomet
SDT	Before sowing of the dazomet treatment
PCK	Primordial stage of the control
PDT	Primordial stage of the dazomet treatment
FCK	Mature fruit body stage of the control
FDT	Mature fruit body stage of the dazomet treatment

### 2.5. High-throughput sequencing

The DNA was extracted with the TGuide S96 Magnetic Soil/Stool DNA Kit (Tiangen Biotech (Beijing) Co., Ltd.) according to manufacturer’s instructions. The DNA concentration of the samples was measured with the Qubit dsDNA HS Assay Kit and Qubit 4.0 Fluorometer (Invitrogen, Thermo Fisher Scientific, Oregon, United States). The 338F: 5′-ACTCCTACGGGAGGCAGCA-3′ and 806R: 5′-GGACTACHVGGGTWTCTAAT-3′ universal primer set was used to amplify the V3-V4 region of the 16S rRNA gene. The ITS1F: 5′-CTTGGTCATTTAGAGGAAGTAA-3′ and ITS2: 5′-GCTGCGTTCTTCATCGATGC-3′ universal primer set was used to amplify the ITS1 region of the ITS gene. Both the forward and reverse primers were tailed with sample-specific Illumina index sequences to allow for deep sequencing. The total PCR amplicons were purified with Agencourt AMPure XP Beads (Beckman Coulter, Indianapolis, IN) and quantified using the Qubit dsDNA HS Assay Kit and Qubit 4.0 Fluorometer (Invitrogen, Thermo Fisher Scientific, Oregon, United States). After the individual quantification step, amplicons were pooled in equal amounts. For the constructed library, an Illumina NovaSeq 6000 (Illumina, Santiago CA, United States) was used for sequencing.

### 2.6. Bioinformatic analysis

The bioinformatics analysis of this study was performed with the aid of the BMK Cloud (Biomarker Technologies Co., Ltd., Beijing, China). According to the quality of single nucleotides, raw data were primarily filtered by Trimmomatic (version 0.33) (Edgar, 2013). Identification and removal of primer sequences was performed by Cutadapt (version 1.9.1) (Callahan et al., 2016). PE reads obtained from previous steps were assembled by USEARCH (version 10) (Segata et al., 2011), followed by chimera removal using UCHIME (version 8.1) (Quast et al., 2012). The high-quality reads generated from the above steps were used in the following analysis. Sequences with similarity ≥97% were clustered into the same operational taxonomic unit (OTU) by USEARCH (v10.0) (Edgar, 2013), and the OTUs with relative abundance <0.005% were filtered. Taxonomy annotation of the OTUs was performed based on the Naive Bayes classifier in QIIME2 (Bolyen et al., 2019) using the SILVA database (release 132) (Quast et al., 2012) with a confidence threshold of 70%. The alpha diversity was calculated and displayed by QIIME2 and R software. Beta diversity was determined to evaluate the degree of similarity of microbial communities from different samples using QIIME. Principal coordinate analysis (PCoA), heatmaps, UPGMA and nonmetric multidimensional scaling (NMDS) were used to analyze the beta diversity. Furthermore, we employed linear discriminant analysis (LDA) effect size (LEfSe) (Segata et al., 2011) to test the significant taxonomic differences among groups. A logarithmic LDA score of 4.0 was set as the threshold for discriminative features.

## 3. Results

### 3.1. Effects of dazomet treatment on the fructification of morel

**Figure 1:**
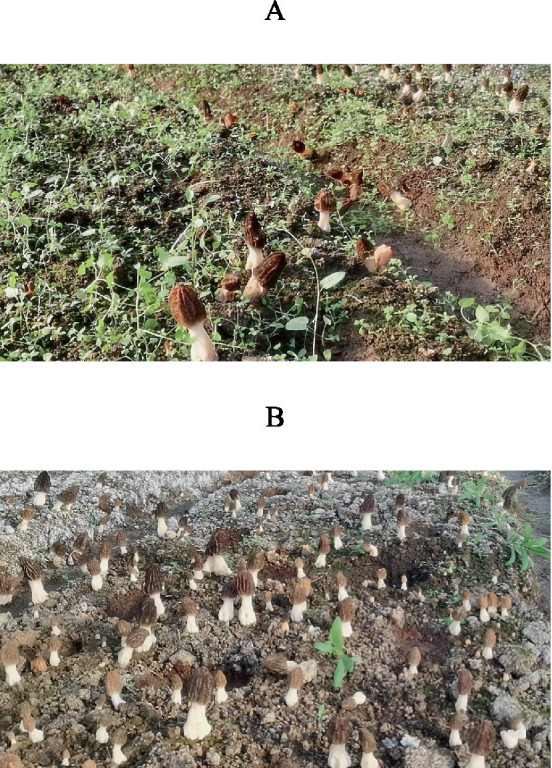
Morel fructification of Qianxi experimental sites. **(A)** Morel fructification of the control. **(B)** Morel fructification of the dazomet treatment.

After 7 days of sowing, morel mitospores appeared on the soil surface. The number of mitospores was not significantly different between the control and dazomet treatments. After irrigation, the primordia formed. Compared with the control, the number of primordia and fruiting bodies in the dazomet treatment was significantly higher, and their distribution was more uniform (Figure 1). The sizes and growth rates of the fruiting bodies in the two treatments were similar. There were many weeds in the control, whereas there were almost no weeds in the dazomet treatment, which may be related to the herbicide effect of dazomet (Brosnan and Breeden, 2009; Jeffries et al., 2017). No obvious diseases or pests were found in the control and dazomet treatments.

**Figure 2:**
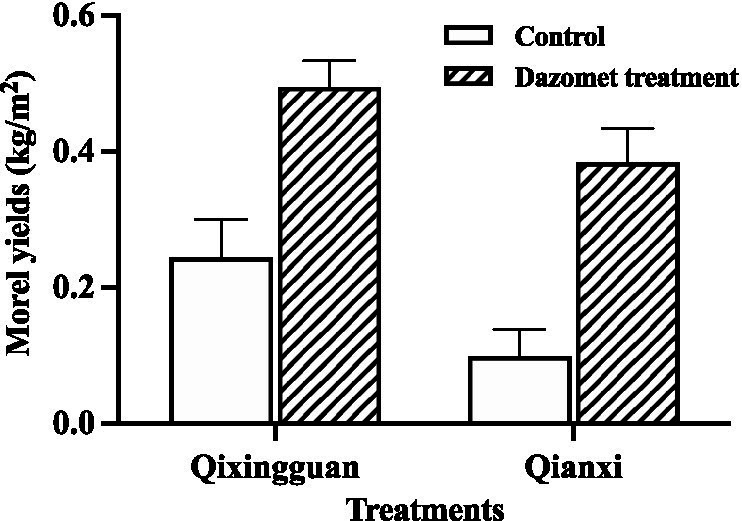
The maximum yield of the control without fumigation with dazomet. The Qixingguan experimental site has been cultivated for 1 year, and the Qianxi experimental site has been cultivated continuously for 2 years. Three independent biological replicates are conducted. Error bars indicate the SD (*n* = 3).

The maximum yield was calculated by fresh weight. Compared with the control, the yield of fruiting bodies in the dazomet treatment was significantly higher. At the Qixingguan experimental site, which is the second year for continuous cultivation of morel, the yield of the control without fumigation with dazomet was 0.24 kg/m^2^, and the yield of the dazomet treatment was twice that of the control. At the Qianxi experimental site, which was undergoing the third year for continuous cultivation of morel, the yield of the control was 0.10 kg/m^2^, and the yield of the dazomet treatment was 3 times that of the control (Figure 2). According to the recollection of the farmers, the morel yield in the first year at the Qixingguan experimental site was approximately 0.33 kg/m^2^. The maximum yields in the first year and second year at the Qianxi experimental site were approximately 0.48 kg/m^2^ and 0.15 kg/m^2^, respectively. The results showed that the morel yield decreased year by year under continuous cropping without appropriate treatment. However, the dazomet treatment increased the maximum yield under continuous cropping, which was close to that of the first year.

### 3.2. High-throughput sequencing results

To understand the mechanism of increasing the morel yield under continuous cropping by fumigation with dazomet, the effect of dazomet treatment on the soil microbial community of the Qianxi experimental site was studied. As described in Table 1, soils were sampled at the sowing, primordium and mature fruit body stages. DNA was extracted from soil samples, and the 16S rDNA V3/V4 region of prokaryotic communities and ITS1 of fungal communities were amplified and sequenced. After quality filtering, a total of 1,082,233 reads for 16S rDNA V3/V4 with a read length of mainly 400–450 bp and 1,384,777 reads for ITS1 with a read length of mainly 220–430 bp across 18 samples were obtained. The rarefaction curves of all samples gradually flattened, which indicated that the sequenced depths were sufficient to reflect the diversity of the samples. After denoising the sequences, 2,305 OTUs for prokaryotic communities and 948 OTUs for fungal communities were generated (Supplementary materials 1, 3). The raw data of 16S rDNA and ITS were uploaded to the Sequence Read Archive (SRA) database of NCB (Accession PRJNA961658).

**Figure 3:**
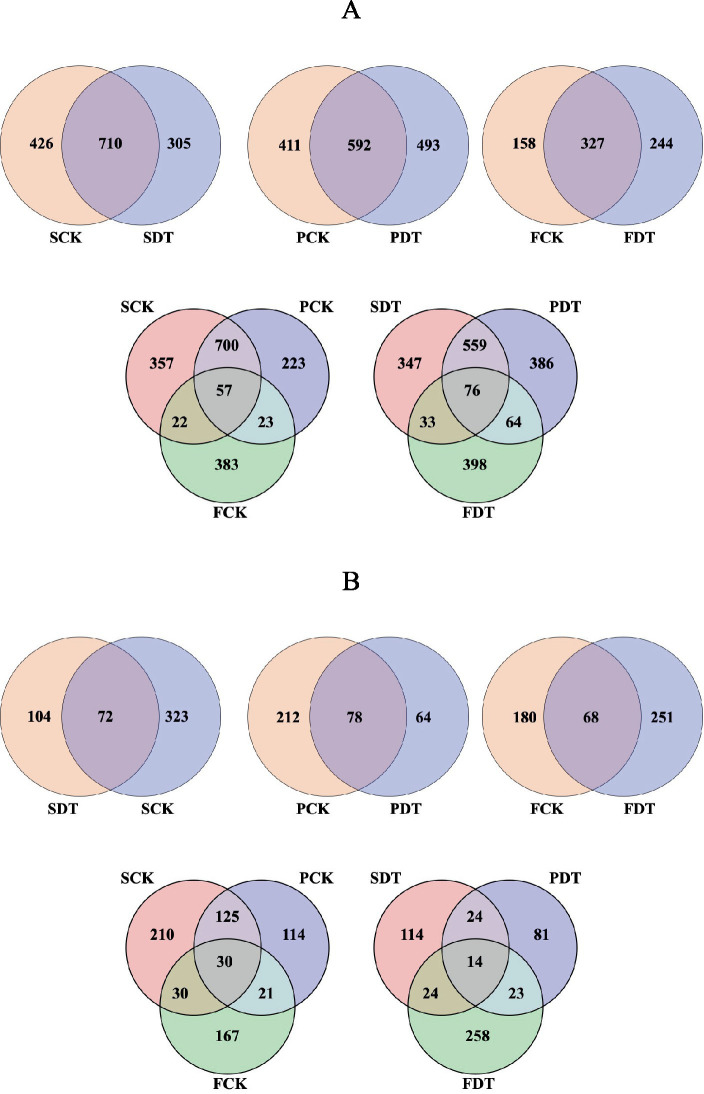
Venn diagram of bacterial OTUs **(A)** and fungal OTUs **(B)**.

Venn diagrams of bacterial OTUs are displayed in Figure 3A. A total of 710 OTUs were found to be common to SDT and SCK, with an additional 305 and 426 OTUs exclusive to SCK and SDT. Fifty-seven OTUs were found to be associated with each stage of the control, with an additional 700, 23 and 22 OTUs common to SCK-PCK, PCK-FCK and FCK-SCK, respectively. Venn diagrams of fungal OTUs are displayed in Figure 3B. Seventy-two OTUs were found to be common to SDT and SCK, with an additional 104 and 323 OTUs exclusive to SDT and SCK. Thirty OTUs were found to be associated with each stage of the control, with an additional 125, 21 and 30 OTUs common to SCK-PCK, PCK-FCK and FCK-SCK, respectively. The fungal OTUs in common between SDT and SCK were at 14.29%, which is far less than the overlap of bacteria (49.27%). This indicates that the influence of dazomet treatment before sowing on fungal OTUs is far greater than on bacterial OTUs.

### 3.3. Alpha diversity analysis of bacterial and fungal communities

**Figure 4:**
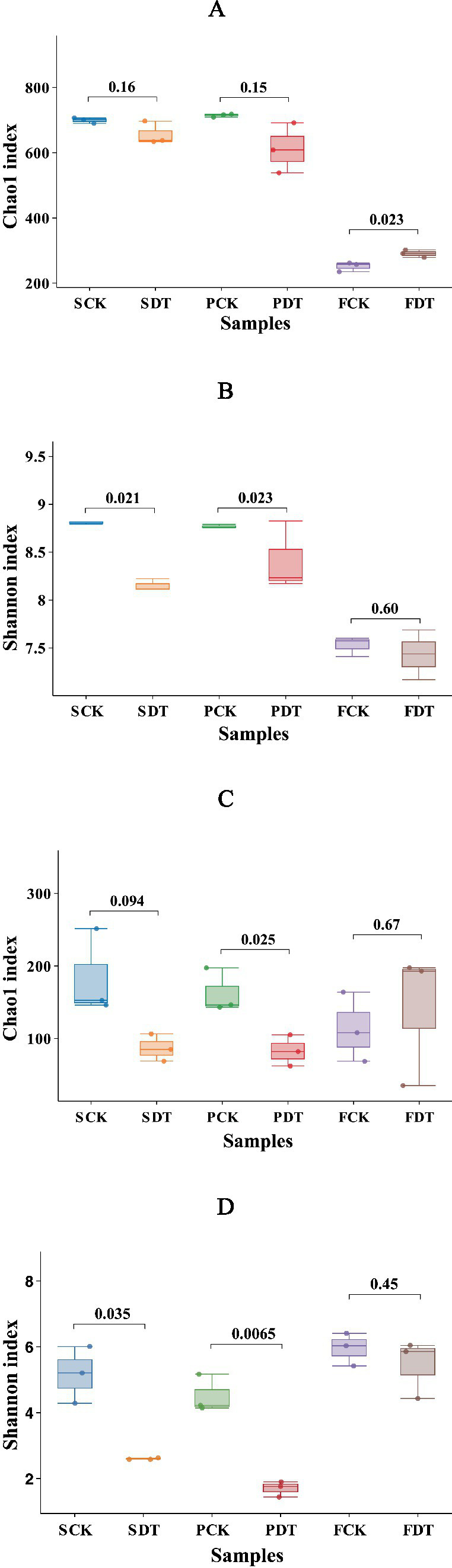
The alpha diversity indices of the samples. **(A)** The bacterial Chao index; **(B)** The bacterial Shannon diversity index; **(C)** The fungal Chao index; **(D)** The fungal Shannon diversity index.

The bacterial alpha diversity Chao1 indices of SCK, SDT, PCK, and PDT were predominantly 500–700, and the Chao1 indices of FCK and FDT were predominantly 250–300 (Figure 4A). The Chao indices of SCK and SDT were not significantly different (*p* = 0.16), but the Shannon index of SDT was significantly lower than that of SCK (*p* = 0.0021) (Figure 4B). This indicates that dazomet treatment before sowing changed the relative abundance rather than the richness of the bacterial community. Compared with those of SCK, SDT, PCK and PDT, the Chao1 and Shannon indices of FCK and FDT were significantly lower. Except for in the dazomet treatment, the cultivation stages also influenced the bacterial community.

For the fungal alpha diversity, the Chao and Shannon indices of SDT were lower than those of SCK, and the patterns of PCK and PDT were similar (Figures 4C,D). This indicates that dazomet treatment before sowing changed both the relative abundance and richness of the fungal community, and the impact of dazomet treatment on the fungal community held to the primordial stage. However, the pattern of the Chao and Shannon indices was remodeled at the fruiting body stage.

### 3.4. Beta diversity analysis of the prokaryotic and fungal communities

**Figure 5:**
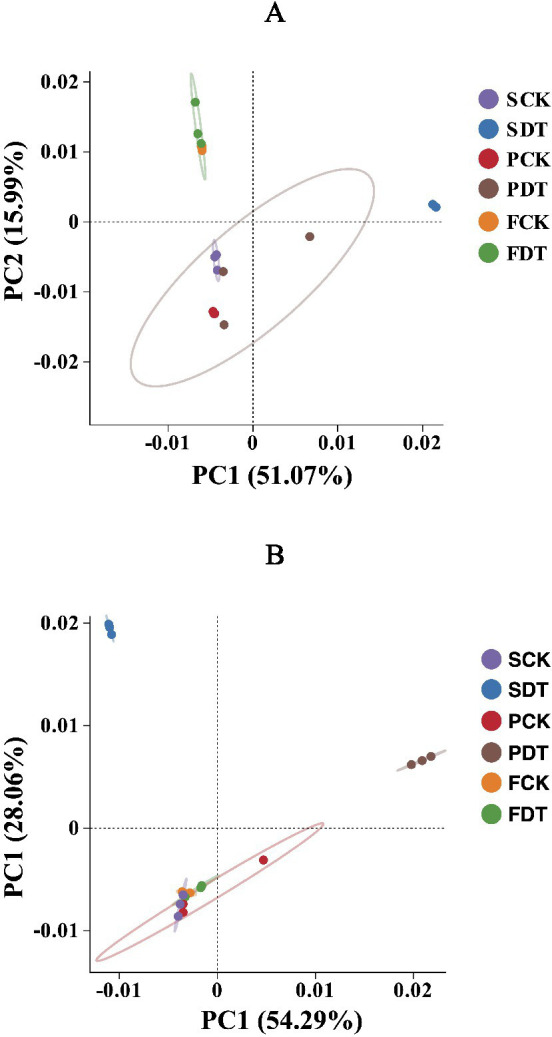
Principal component analysis of the prokaryotic community **(A)** and fungal community **(B)**. Each dot represents a sample. The confidence ellipse defines the region that contains 95% of all samples that can be drawn from the underlying Gaussian distribution.

Beta diversity analysis was processed by QIIME software to compare species diversity between different samples. In general, the distance between two dots (samples) indicates similarity between two samples in species composition. The explanatory values of PC1 and PC2 in the bacterial community were 51.07 and 15.99%, respectively (Figure 5A). The PCA grouped 18 samples into 3 clusters by a 95% confidence ellipse. Except for SCK, the clustering of other samples correlated with the cultivation stages of morel. SCK was clustered with PCK and PDT rather than SDT, indicating that SCK contained more bacterial species consistent with those of PCK and PDT. It is inferred that those consistent bacteria may be closely associated with morel cultivation and may have remained from morel cultivation in the last year. SDT formed an independent cluster far from SCK, which shows that dazomet treatment significantly changed the composition of the bacterial community.

The explanatory values of PC1 and PC2 in the fungal community were 54.29 and 28.06%, respectively (Figure 5B). The PCA grouped the 18 samples into 3 clusters by a 95% confidence ellipse. SDT and PDT formed two clusters independently, and SCK, PCK, FCK, and FDT formed a cluster. SDT was far from SCK, indicating that dazomet treatment significantly changed the composition of the fungal community. The distance of PDT from PCK may be the result of the difference between SCK and SDT. The convergence of FDT and FCK may be related to the influence of the morel cultivation stage on the fungal community. SCK, PCK, FCK and FDT shared a common fungal community, which suggests that SCK and PCK retained the fungal community enriched by morel cultivation the previous year.

### 3.5. Relative abundance of major bacterial and fungal phyla

**Figure 6:**
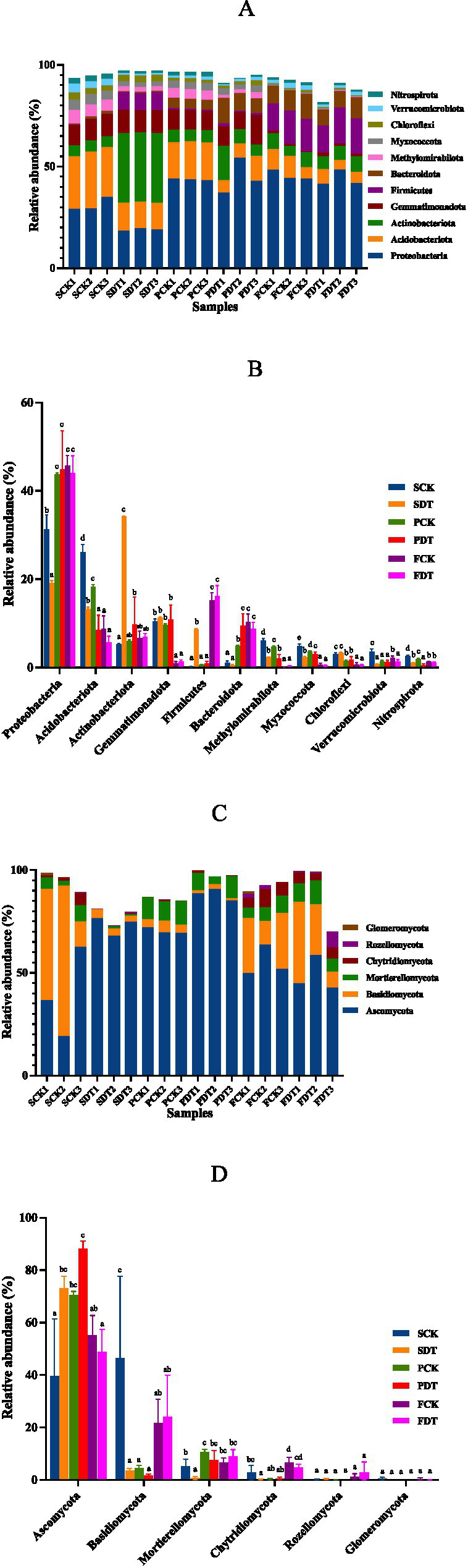
The relative abundance of major bacterial and fungal phyla in samples. **(A)** Cumulative relative abundance of bacterial phyla >2% in at least one sample. **(B)** ANOVA of the relative abundance of bacterial phyla. **(C)** Cumulative relative abundance of fungal phyla >1% in at least one sample. **(D)** ANOVA of the relative abundance of fungal phyla.

The bacterial phyla with relative abundances >2% are displayed in Figures 6A,B. Proteobacteria (31.22% ± 2.73%), Acidobacteriota (26.14% ± 1.39%) and Gemmatimonadota (10.50% ± 0.40%) were the dominant bacterial phyla in the SCK. Compared with those of SCK, the relative SDT abundances of Proteobacteria, Acidobacteriota, Methylomirabilota, Myxococcota, Verrucomicrobiota and Nitrospirota increased significantly, and the relative abundances of Actinobacteriota and Firmicutes in SDT decreased significantly. The results show that dazomet treatment changed the relative abundance of the predominant phyla of the soil. Compared with SCK, FCK significantly changed the relative abundances of all 11 predominant phyla. Compared with SDT, FDT significantly changed the relative abundances of 9 bacterial phyla except Verrucomicrobiota and Nitrospirota. Except for Verrucomicrobiota, FCK and FDT converged in the relative abundance of the other 10 predominant phyla regardless of the difference before the sowing stage. This suggests that morel cultivation also significantly influences the relative abundance of the predominant bacterial phyla.

The fungal phyla with a relative abundance >1% are displayed in Figures 6C,D. Ascomycota (39.56% ± 17.85%) and Basidiomycota (46.53% ± 25.42%) were the dominant fungal phyla in the SCK. Compared with SCK, the relative abundance of Ascomycota was significantly higher, and the relative abundances of Basidiomycota, Mortierellomycota and Chytridiomycota were significantly lower in SDT. This indicates that dazomet treatment also changed the fungal community. Compared with SCK, FCK significantly changed the relative abundances of Ascomycota, Basidiomycota, Mortierellomycota and Chytridiomycota. Compared with SDT, FDT also significantly changed the relative abundances of Ascomycota, Basidiomycota, Mortierellomycota and Chytridiomycota. This indicates that morel cultivation also significantly influences the fungal community.

### 3.6. Relative abundance of major bacterial and fungal genera

**Figure 7:**
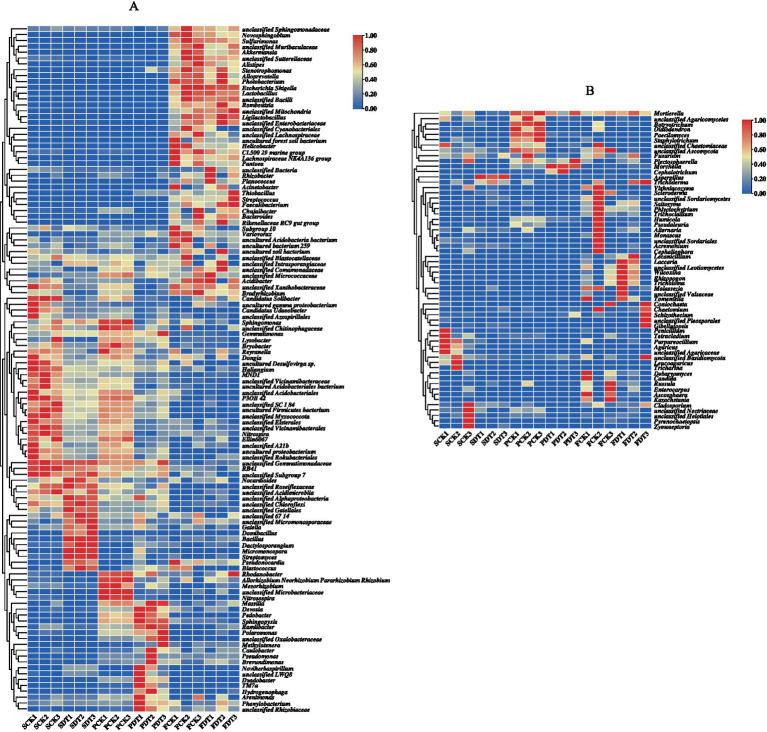
Heatmap of dominant bacterial genera **(A)** and dominant fungal genera **(B)**.

A full list of bacterial OTUs and genera is provided in Supplementary materials 1, 2. The relative abundance of bacterial genera >3% is displayed as a heatmap in Figure 7A. Compared with SCK, the relative abundances of 26 genera, including *Candidatus Udaeobacter*, unclassified *Azospirillales*, *Sphingomonas*, unclassified *Chitinophagaceae*, etc., were significantly lower in SDT. In addition, the relative abundances of those 26 genera in PDT were also significantly lower than those in PCK. This indicates that the influences of dazomet treatment on those 26 genera lasted from the presowing to primordial stages. Compared with SCK, SDT, PCK and PDT, the relative abundances of 34 genera, including unclassified *Sphingomonadaceae*, *Novosphingobium*, *Sulfurimonas*, unclassified *Muribaculaceae*, etc., increases significantly in FCK and FDT. However, the relative abundances of major bacterial genera for FCK and FDT were similar.

A full list of fungal OTUs and genera is provided in Supplementary materials 3, 4. The relative abundance of fungal genera >3% is displayed as a heatmap in Figure 7B. Among 18 samples of 6 treatments, the change in the relative abundance for those 60 dominant genera was not as regular as that of bacteria.

### 3.7. Indicator genera

**Figure 8:**
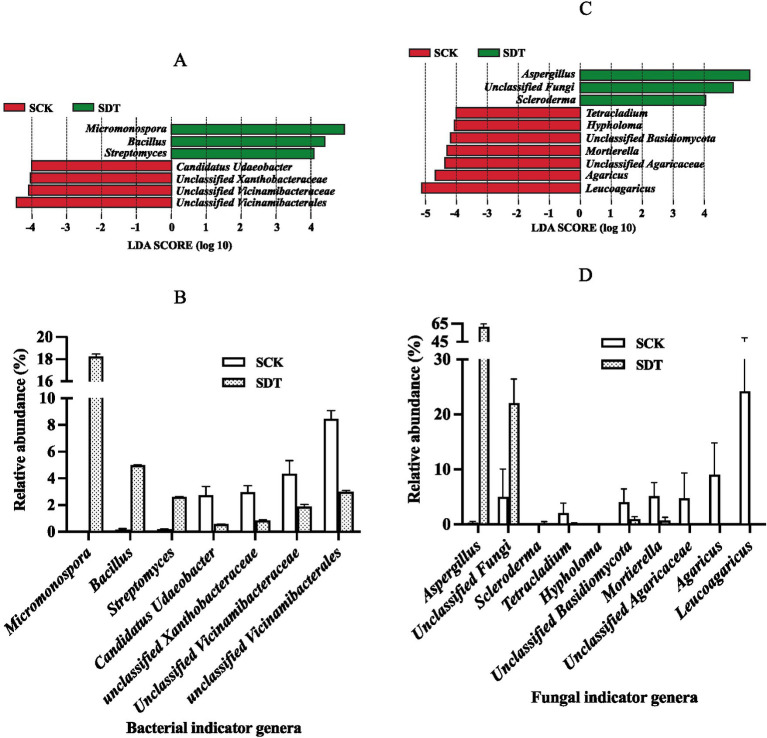
The bacterial and fungal indicator genera of SCK and SDT. **(A)** Bacterial indicator genera. **(B)** The relative abundance of bacterial indicator genera. **(C)** Fungal indicator genera. **(D)** The relative abundance of fungal indicator genera. Three independent biological replicates are conducted. Error bars indicate the SD (*n* = 3).

Linear discriminant analysis (LDA) effect size (LEfSe) was used to find the dominant indicator genera. By comparing SDT and SCK, the bacterial indicator genera of SCK and SDT are displayed in Figure 8A. The shift in the relative abundance of those indicator genera shows that dazomet treatment not only reduces the relative abundance of SCK indicator genera but also increases the relative abundance of SDT indicator genera (Figure 8B). It is interesting that the indictor genera of SDT were beneficial to edible mushrooms because several studies have demonstrated that *Micromonospora*, *Bacillus*, and *Streptomyces* species function in biocontrol, plant growth promotion, and mushroom composting (Sahin, 2005; Hirsch and Valdés, 2010; Sotoyama et al., 2016; Stanojević et al., 2019; Büchner et al., 2022). Because the SCK indicator genera, including *Candidatus Udaeobacter*, Unclassified *Xanthobacteraceae*, Unclassified *Vicinamibacteraceae*, and unclassified *Vicinamibacterales*, could not be identified accurately, their effects on morel growth could not be determined.

The fungal indicator genera of SCK and SDT are displayed in Figures 8C,D. *Aspergillus* was the fungal indicator genus with the highest relative abundance of 61.33% in SDT, and *Leucoagaricus*, *Agaricus*, and *Mortierella* were the fungal indicator genera in SCK.

### 3.8. Relative abundance of morel

**Figure 9:**
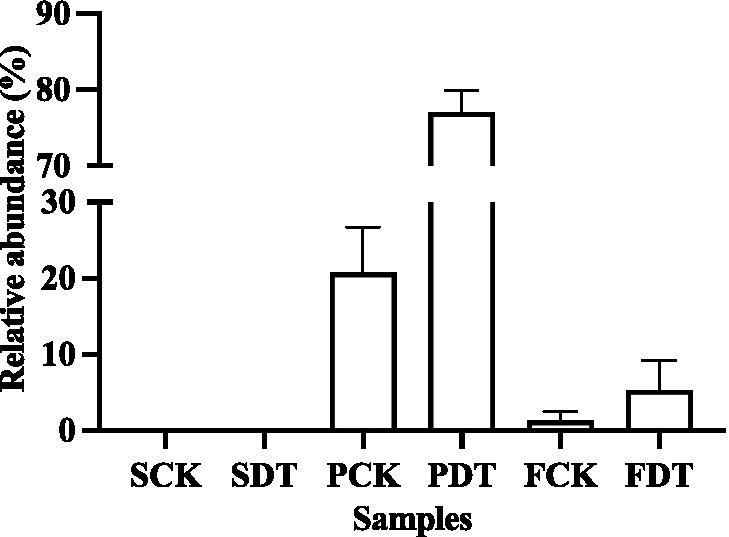
The relative abundance of morel in soil samples. Three independent biological replicates are conducted. Error bars indicate the SD (*n* = 3).

Morels can produce a large number of fruiting bodies only when they form enough vegetative mycelia. In this study, the relative abundance of morel in soil was analyzed. Morel was not detected in SCK and SDT, indicating that the mycelium of the morel of the last year had completely disappeared before sowing in the current year. The relative abundance of morel was highest at the primordium stage, and it decreased significantly as the fruit body grew and developed. In PCK and PDT, the number of conidia that formed on the soil surface was similar, while the relative abundance of morel in PDT was evidently higher than that in PCK (Figure 9). This indicates that dazomet treatment before sowing improved morel mycelial growth in soil. Therefore, the decrease in the number of primordia and fruiting bodies may be related to the decrease in the relative abundance of morel in soil.

## 4. Discussion

In this study, we found that dazomet treatment changed soil microbial communities and significantly increased yield under continuous cropping. This result suggested that the obstacle of continuous cropping in morels is closely related to the soil microbial communities.

### 4.1. The decrease in the relative abundance of soil-borne fungal pathogens

Many continuous cropping obstacles are associated with the accumulation of soil-borne pathogens. Soil-borne pathogens, including *Fusarium*, *Pythium*, *Rhizotonia*, *Cylindrocarpon*, and *Phytophthora*, arising from continuous cropping, cause diseases such as root rot, damping-off and wilt that have a direct cost to crop growth, survival and yield (Ampt et al., 2019; Dignam et al., 2022). For example, *Fusarium oxysporum* is a well-known soilborne plant pathogen that causes severe vascular wilt in economically important crops such as American ginseng, tobacco, watermelon and strawberry (Koike and Gordon, 2015; Lamondia, 2015; Li C. et al., 2021; Wu et al., 2022).

It has been reported that fungi, including *Penicillium*, *Trichoderma*, *Aspergillus*, *Fusarium*, *Botrytis*, and *Clonostachys*, increase in abundance under continuous cropping of morel, and that these fungi may be the main pathogens that cause a reduction in production for continuous *M. sextelata* cultivation (Liu et al., 2022). Nonfructification is sometimes encountered in large-scale morel farming for unknown reasons. It has been suggested that the soils with successful fructification have significantly higher diversity in both the fungal and bacterial communities than those with nonfructification, and most nonfructification soils have been shown to be dominated by a high proportion of certain fungal genera, typically *Acremonium*, *Mortierella*, and *Paecilomyces* (Tan et al., 2021). *Penicillium*, *Trichoderma*, *Aspergillus*, *Fusarium*, *Botrytis*, *Clonostachys*, *Acremonium*, *Mortierella*, and *Paecilomyces* are pathogenic fungi in the production of edible mushrooms and plants. Notably, the pathogenicity of only a few pathogens on morel has been confirmed and reported. Typical diseases include stipe rot disease caused by the *Fusarium incarnatum* – *F. equiseti* species complex (Guo et al., 2016), pileus rot disease caused by *Diploöspora longispora* (He et al., 2018; Shi et al., 2022; Sun et al., 2023), white mold disease caused by *Paecilomyces penicillatus* (He et al., 2017; Fu et al., 2022), cobweb disease caused by *Cladobotryum protrusum* (Lan et al., 2020), white mildew disease caused by *Aspergillus* sp. (Yu et al., 2020). To the best of our knowledge, none of these soil-borne diseases has been proven to be directly related to the continuous cropping of morel.

**Figure 10:**
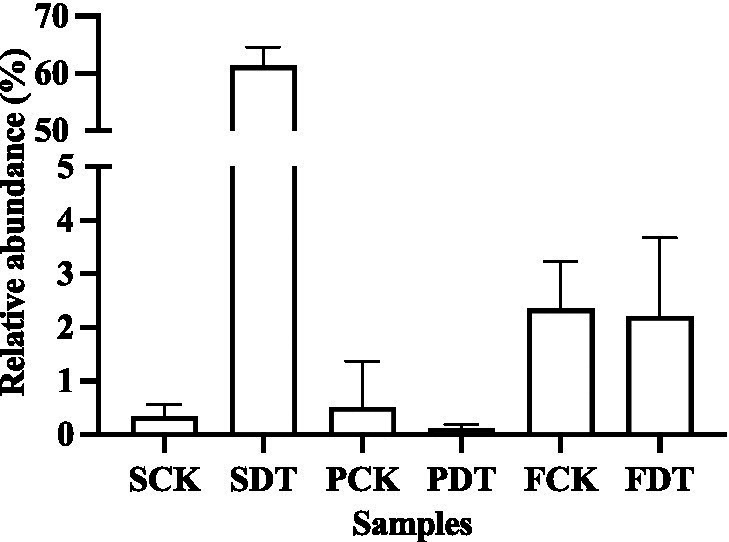
The relative abundance of *Aspergillus* in soil samples. Three independent biological replicates are conducted. Error bars indicate the SD (*n* = 3).

**Figure 11:**
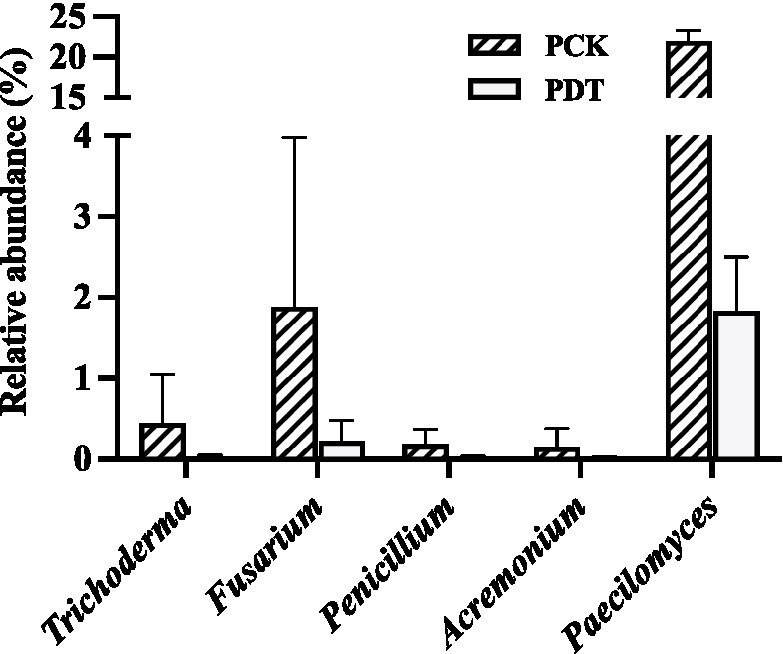
The relative abundance of *Trichoderma*, *Fusarium*, *Penicillium*, *Acremonium* and *Paecilomyces* in PCK and PDT. Three independent biological replicates are conducted. Error bars indicate the SD (*n* = 3).

In this study, the relative abundance of *Aspergillus* was only 0.3% in SCK. Dazomet treatment increased the relative abundance of *Aspergillus* to 61.3%, which made *Aspergillus* the indicator genus of SDT. After inoculation with morel culture, the relative abundance of *Aspergillus* decreased to 0.1% in PDT and 2.2% in FDT (Figure 10). No white mildew disease was found in the dazomet treatment, which indicates that the effect of *Aspergillus* on morel warrants further study. The relative abundance of *Paecilomyces* was dynamic during morel cultivation, and the highest abundance was at the primordial phase (Figure 7B). The relative abundance of *Paecilomyces* in PCK was 21.9% and that in PDT was 1.8% (Figure 11), which showed that dazomet treatment had a significant effect on the relative abundance of *Paecilomyces*. The main symptom of obstacles associated with continuous cropping of morel is that the number of primordia and fruiting bodies is reduced. However, no obvious white mold disease was found in this study. Perhaps the primordium was too small to make the infection undetectable. It has been reported that once the very young and small morel fruiting bodies are infected by *Paecilomyces*, they stop growing (He et al., 2017). *Trichoderma*, *Fusarium*, *Penicillium*, and *Acremonium* were detected in the control and dazomet treatments. Similar to that of *Paecilomyces*, the relative abundance of these four fungi in PDT was significantly lower than that in PCK as a result of dazomet treatment (Figure 11). It has been reported that Gibberella, Microidium, Sarocladium and Streptomyces accounted for a high proportion in soils with low or no morel yield (Yu et al., 2022). However, those fungi were not detected in this study. In addition, no fungal pathogens *Diploöspora* and *Cladobotryum* were detected in this study. Therefore, the increase in yield by dazomet treatment may be closely related to the decrease in the abundance of *Paecilomyces*, *Trichoderma*, *Fusarium*, *Penicillium*, and *Acremonium*.

### 4.2. The increase in the relative abundance of soil-borne beneficial bacteria

A wide variety of interactions between bacteria and cultivated mushrooms have been described, leading to positive effects on edible mushrooms (Frey-Klett et al., 2011; Carrasco and Preston, 2020; Li et al., 2022). For example, *Pseudomonas* triggers primordium formation in *Agaricus bisporus* by removing inhibitory C8 compounds produced by the mycelium (Noble et al., 2009). *Bacillus* can substantially improve the growth and yield of some edible mushrooms by inhibiting the pathogenic fungi *Trichoderma harzianum* and *Fusarium oxysporum* (Velázquez-Cedeño et al., 2008; Sarwar et al., 2018). The continuous cropping obstacle of *Ganoderma lingzhi* may be related to the decline in the relative abundance of beneficial bacteria such as *Sphingomonas*, *Anaeromyxobacter*, *Bradyrhizobium* and *Dehalococcoides* in the covering soil (Yuan et al., 2021).

The morel can farm *Pseudomonas putida*, including bacterial dispersal, bacterial rearing with fungal exudates, and harvesting and translocation of bacterial carbon. At the same time, *P. putida* can stimulate the formation of sclerotia and improve the stress resistance of morel mycelium (Pion et al., 2013). Additionally, *Pseudomonas* can increase the hydrolysis of organic nitrogen sources by enhancing the activity of proteolytic enzymes produced by the morel and improve biomass for both partners (Lohberger et al., 2019). *Bacillius* was found to substantially affect the growth and development of morel fruiting bodies in recent studies (Longley et al., 2019; Zhang et al., 2019; Liu et al., 2022). *Paenibacillus* may play similar roles to *Bacillus* in morel cultivation (Longley et al., 2019). In addition, some noteworthy bacterial microbes involved in nitrogen fixation and nitrification, such as *Arthrobacter*, *Bradyrhizobium*, *Devosia*, *Pseudarthrobacter*, *Pseudolabrys*, and *Nitrospira*, have been identified in soils with high morel yields (Yu et al., 2022).

**Figure 12:**
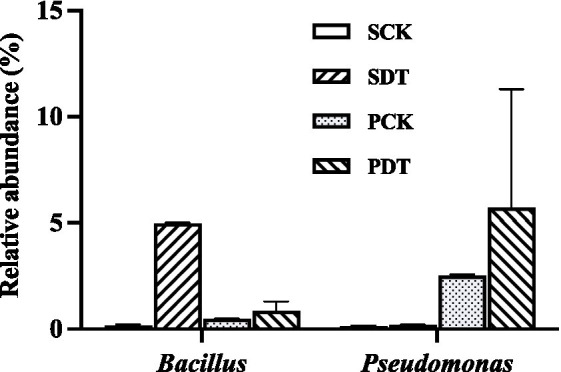
The relative abundance of *Bacillus* and *Pseudomonas* in SCK, SDT, PCK, and PDT. Three independent biological replicates are conducted. Error bars indicate the SD (*n* = 3).

In this study, the relative abundance of *Bacillus* in SDT increased by 32 times compared with that in SCK, and the relative abundance of *Bacillus* in PDT was 79% higher than that in PCK. Similarly, in SDT and PDT, the relative abundance of *Pseudomonas* was significantly higher than that in SCK and PCK, respectively (Figure 12). In addition, *Paenibacillus*, *Devosia*, and *Nitrospira* were also detected in this study, but they were not the dominant genera in the samples (Supplementary material 2). No *Arthrobacter*, *Bradyrhizobium*, *Pseudarthrobacter*, or *Pseudolabrys* were detected in this study. The results showed that dazomet treatment increased the relative abundance of some beneficial bacteria, such as *Bacillus* and *Pseudomonas*, in the soil.

### 4.3. Restoration of the soil microbial community

Soil microbial diversity is important to sustainable agriculture because microbes can mediate many biochemical processes that support agricultural production. These processes include recycling of plant nutrients, maintenance of soil structure and degradation of agrochemicals (Qin et al., 2017). Soil microbial community structure affects crop health and can also be used as an indicator of soil health (Chen Y. et al., 2022). To date, a number of studies have shown that continuous cropping disrupts the soil microbial community composition (Wu et al., 2022). For instance, the bacterial and fungal diversities were observably altered after the long-term monoculture of *Ganoderma lingzhi* (Yuan et al., 2019, 2021).

As in previous studies (Liu et al., 2017; Benucci et al., 2019; Longley et al., 2019; Orlofsky et al., 2021), the soil microbial communities in this study were dynamic during the cultivation of morel for the control and dazomet treatments. After the long-term monoculture of morel, the disruption of the soil microbial community increases. Soil microbial community disruption negatively affects fructification (Liu et al., 2022; Yu et al., 2022). It has been reported that morel fructification in large-scale cultivation is positively correlated with the diversity and evenness of soil microbial communities (Tan et al., 2021). The principal component analysis (PCA) of bacterial communities showed that SCK, PCK and PDT belonged to one ellipse by 95% confidence, whereas SDT formed an independent cluster far away from the others (Figure 5A). Similar results were obtained for the PCA of the fungal communities (Figure 5B). This result suggested that dazomet treatment partially restored the bacterial and fungal communities from those in continuous cropping soil. Therefore, the increase in yield by dazomet treatment may also be associated with the recovery of microbial communities in continuous cropping soil.

It is well known that rotation with paddy rice can also increase morel yield under continuous cropping. Anaerobic conditions lead to the domination of anaerobic microbes and result in significant changes in soil pH, metal ion availability, and microbial community composition. These changes have negative impacts on soil-borne fungal pathogens (Momma et al., 2013; Khadka and Miller, 2021). In nature, the morel reproduces prolifically in the first year following fires, after which the population rapidly declines (Masaphy and Zabari, 2013; Larson et al., 2016; Miller et al., 2017). This phenomenon may also be related to the soil microbial community, which can be altered by wildfire and stimulates fructification. As mentioned above, the methods to increase the fructification of morel, including dazomet fumigation, rotation with paddy rice, and postfire treatment, could change the soil microbial community. This further suggests the relationship between the obstacle of continuous cropping in morels and soil microbial communities.

**Figure 13:**
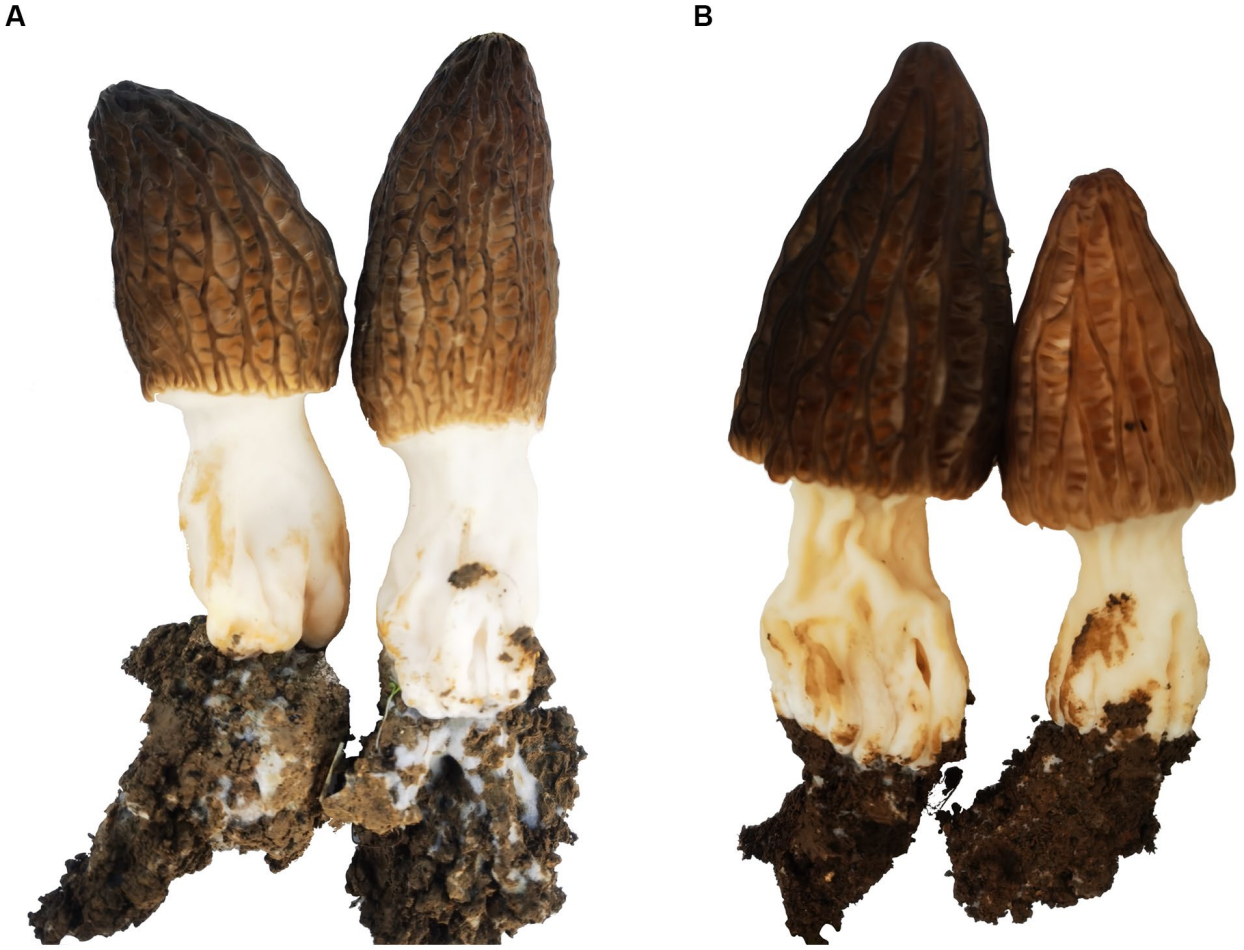
Morel fruit bodies cultivated under noncontinuous cropping **(A)** and under continuous cropping **(B)**.

Although dazomet fumigation can increase morel yield, the continuous cropping obstacle has not been completely eliminated. We found that there were abundant white hyphae at the bottom of the fruiting bodies growing in the soil for the first time, whereas there was only a small amount of hyphae at the bottom of the fruiting body growing in the continuous cropping soil (Figure 13). The bottoms of the fruiting bodies growing in the soil fumigated with dazomet were similar to those growing in the continuous cropping soil. To completely eliminate continuous cropping obstacles and improve the yield of morel, further research is needed.

## 5. Conclusion

Our results suggest that dazomet fumigation before inoculation of morel culture decreased the relative abundance of soil-borne fungal pathogens, including *Paecilomyces*, *Trichoderma*, *Fusarium*, *Penicillium*, and *Acremonium*. On the other hand, dazomet treatment increased the relative abundance of beneficial soil bacteria, including *Bacillius* and *Pseudomonas*, which positively affected the growth of mycelia and fructification of the morel. Alpha diversity and beta diversity analysis results showed that dazomet treatment altered the bacterial and fungal communities in continuous cropping soil. The decrease in soil-borne fungal pathogens, the increase in beneficial bacteria and the recovery of the microbial community increased the abundance of morel mycelium, improved the number of primordia and fruiting bodies, and enhanced the yield of the morel under continuous cropping conditions. In summary, dazomet treatment can partially eliminate obstacles associated with continuous cropping and can improve the yield of the morel.

## Data availability statement

The datasets presented in this study can be found in online repositories. The names of the repository/repositories and accession number(s) can be found below: BioProject accession number: PRJNA961658.

## Author contributions

BC conducted the experiments. GS, TZ, QF, NY, MC, and JZ analyzed the data and wrote the manuscript. XW and BZ reviewed the manuscript. RZ designed the research and supervised the work. All authors contributed to the article and approved the submitted version.

## Funding

This work was supported by the Chinese Agriculture Research System (CARS-20); Fundamental Research Funds for Central Nonprofit Scientific Institution (No. 1610132021007); Guizhou Provincial Key Technology R&D Program ([2021]YB201, [2022]YB083, [2023]YB054); Guizhou Provincial Major Scientific and Technological Program ([2019]3007) and Guizhou Province Science and Technology Innovation Ability Pro-motion Special Project ([2021]1).

## Conflict of interest

The authors declare that the research was conducted in the absence of any commercial or financial relationships that could be construed as a potential conflict of interest.

## Publisher’s note

All claims expressed in this article are solely those of the authors and do not necessarily represent those of their affiliated organizations, or those of the publisher, the editors and the reviewers. Any product that may be evaluated in this article, or claim that may be made by its manufacturer, is not guaranteed or endorsed by the publisher.
